# Infrapatellar Fat Pad Stem Cells Responsiveness to Microenvironment in Osteoarthritis: From Morphology to Function

**DOI:** 10.3389/fcell.2019.00323

**Published:** 2019-12-10

**Authors:** Elena Stocco, Silvia Barbon, Monica Piccione, Elisa Belluzzi, Lucia Petrelli, Assunta Pozzuoli, Roberta Ramonda, Marco Rossato, Marta Favero, Pietro Ruggieri, Andrea Porzionato, Rosa Di Liddo, Raffaele De Caro, Veronica Macchi

**Affiliations:** ^1^Department of Neurosciences, Institute of Human Anatomy, University of Padova, Padua, Italy; ^2^LifeLab Program, Consorzio per la Ricerca Sanitaria (CORIS), Padua, Italy; ^3^Foundation for Biology and Regenerative Medicine, Tissue Engineering and Signaling ONLUS, Padua, Italy; ^4^Department of Pharmaceutical and Pharmacological Sciences, University of Padova, Padua, Italy; ^5^Musculoskeletal Pathology and Oncology Laboratory, Department of Surgery, Oncology and Gastroenterology DiSCOG, University of Padova, Padua, Italy; ^6^Department of Orthopaedics and Orthopaedic Oncology, University of Padova, Padua, Italy; ^7^Rheumatology Unit, Department of Medicine – DIMED, University Hospital of Padova, Padua, Italy; ^8^Clinica Medica 3, Department of Medicine – DIMED, University of Padova, Padua, Italy

**Keywords:** infrapatellar fat pad, osteoarthritis, stem cells, inflammation, reprograming

## Abstract

Recently, infrapatellar fat pad (IFP) has been considered as a source of stem cells for cartilage regeneration in osteoarthritis (OA) due to their ability for differentiation into chondrocytes. However, stressful conditions, like that related to OA, may induce a pathogenic reprograming. The aim of this study was to characterize the structural and functional properties of a new population of stem cells isolated from osteoarthritic infrapatellar fat pad (OA-IFP). Nine OA patients undergoing total knee arthroplasty (TKA) were enrolled in this study [median age = 74 years, interquartile range (IQR) = 78.25-67.7; median body mass index = 29.4 Kg/m^2^, IQR = 31.7-27.4]. OA-IFP stem cells were isolated and characterized for morphology, stemness, metabolic profile and multi-differentiative potential by transmission electron microscopy, flow cytometric analysis, gene expression study and cytochemistry. OA-IFP stem cells displayed a spindle-like morphology, self-renewal potential and responsiveness (CD44, CD105, VEGFR2, FGFR2, IL1R, and IL6R) to microenvironmental stimuli. Characterized by high grade of stemness (*STAT3*, *NOTCH1, c-Myc*, *OCT-4, KLF4*, and *NANOG*), the cells showed peculiar immunophenotypic properties (CD73^+^/CD39^+^/CD90^+^/CD105^+^/CD44^–/+^/CD45^–^). The expression of HLA-DR, CD34, Fas and FasL was indicative of a possible phenotypic reprograming induced by inflammation. Moreover, the response to mechanical stimuli together with high expression level of *COL1A1* gene, suggested their possible protective response against *in vivo* mechanical overloading. Conversely, the low expression of *CD38/NADase* was indicative of their inability to counteract NAD^+^-mediated OA inflammation. Based on the ultrastructural, immunophenotypic and functional characterization, OA-IFP stem cells were hypothesized to be primed by the pathological environment and to exert incomplete protective activity from OA inflammation.

## Introduction

In osteoarthritis (OA) the clinical signs suggesting the occurrence of an inflammatory process are generally evident. These include manifestations such as swelling, heat, stiffness and pain, which are clearly recognizable since the disease onset and continue along its course (Favero et al., 2015). To date, arthroplasty is considered the only decisive option in OA; the other therapeutic strategies for early OA (e.g., weight reduction, exercise, braces, non-steroidal anti-inflammatory drugs, symptomatic slow-acting drugs, intra-articular injections of glucocorticoid or hyaluronic acid) can only slow its progression without any significant effect on the cartilage integrity (Im, 2018). Worldwide, approximately 9.6% of men and 18% of women aged ≥ 60 years are estimated to have symptomatic OA (Michaud et al., 1996); hence, the extent of the disease, the significant impairment of patients’ quality of life and the related costs require a better characterization of the pathophysiological mechanisms involved in its development. This would ensure more effective therapeutic approaches to OA, overcoming the limitations of the current ones, also suggesting alternative and/or tailored options for disease management.

Currently, it is commonly accepted that OA is not merely a cartilage disease but a whole joint disease involving different structures such as meniscus, synovial membrane, and infrapatellar fat pad (IFP) (Belluzzi et al., 2019). In the recent years, the potential role of the IFP in OA has been suggested by many researchers. For a long time, this peculiar extra-synovial adipose tissue was simplistically retained a deformable space filler endowed with shock absorber properties in the knee joint and a bystander in knee OA; conversely, it is now considered an emerging player in knee OA with a multifunctional role in knee joint homeostasis, also mediating joint pain and inflammation (Ioan-Facsinay and Kloppenburg, 2013; Belluzzi et al., 2017, 2019; Macchi et al., 2018). This occurs through an active secretion of cytokines and adipokines (Ioan-Facsinay and Kloppenburg, 2013) whose levels are gender dependent, being normally higher in women than in men even after adjusting for body mass index (BMI) (Poonpet and Honsawek, 2014). Therefore, it can be assumed that the IFP, similar to subcutaneous and visceral adipose tissue, is an active organ whose products contribute to inflammatory and degenerative processes underlying common joint diseases (Toussirot et al., 2007).

Furthermore, the IFP itself is affected by immune cells in OA; in fact, compared to healthy-IFPs, it shows an increase in inflammatory infiltration, vascularization and fibrosis with thickened interlobular septa also supported by higher levels of VEGF, MCP-1, and IL-6 proteins (Favero et al., 2017; Fontanella et al., 2018).

In addition to constitutive stromal vascular cells represented by hematopoietic cells (i.e., macrophages, T cells, mast cells, and B cells) and endothelial cells, also progenitor cells with mesenchymal stem cell-like characteristics have been recognized in the IFP (Hindle et al., 2017; Huri et al., 2018). Stem cells regenerate tissues in homeostasis but they also sense and respond to stressful conditions (Naik et al., 2018) undergoing a possible pathogenic reprograming under long-lasting inflammation (Michael et al., 2016). Thus, the purpose of this study was to isolate OA-IFP stem cells from patients undergoing total knee arthroplasty (TKA) and provide a broad characterization of such cell populations. A better understanding of their origin and pathophysiological role in OA may suggest a possible novel interpretive key to explain histopathological features commonly encountered in OA joints.

## Materials and Methods

### Patients

This study was performed after receiving institutional review board approval (CESC Code: 4510/AO/18). Patients affected by OA undergoing to TKA, showing good health, without tumor and/or knee comorbidities and not suffering from infective conditions were eligible. Nine patients were enrolled in this study after signing the informed consent [median age = 74 years, interquartile range (IQR) = 78.25-67.7; median body mass index = 29.4 Kg/m^2^, IQR = 31.7-27.4]; demographic data are reported in Table 1. At the moment of sample collection, the patients were naïve from therapies. Tissue specimens were collected during TKA and then rapidly processed for the isolation of stromal cells.

**TABLE 1:** Patient’s demographic data.

**Patient number**	**Sex**	**Age, years**	**BMI, Kg/m^2^**	**Kellgren Lawrence Scale**	**Comorbidities**
1	F	68	29.4	3	H
2	F	67	27.7	3	H
3	F	74	30.1	3	H, D
4	M	64	27.4	3	H, D2
5	M	73	36.3	4	H, D2
6	F	79	24.5	3	H, D, D2
7	F	76	27.3	3	H, D
8	M	79	32.3	3	H, D
9	F	78	31.6	3	H, D

*BMI, body mass index; F, female; M, male; H, hypertension; D, dyslipidemia; D2, diabetes type 2.*

### Cell Isolation

Osteoarthritic infrapatellar fat pad stem cells were isolated according to a modified protocol by Hindle et al. (2017). After washing tissues in phosphate buffered saline (PBS) supplemented with 2% penicillin/streptomycin (P/S; Sigma-Aldrich), enzymatic digestion was performed overnight at 37°C in a medium consisting of Dulbecco’s Modified Eagle Medium High Glucose (DMEM HG, Gibco, Thermo Fisher Scientific, Waltham, MA, United States), 1% collagenase B (Roche, Basel, Switzerland) and 1% P/S. The cell suspension was filtered through 40-μm cell strainer (BD Falcon, Franklin Lakes, NJ, United States) and centrifuged before seeding the pellet in proliferation medium (DMEM HG, 10% FBS, 1% P/S).

### Long-Term Proliferative Potential and Metabolic Activity

The proliferation rate of OA-IFP stem cells was evaluated from 8th to 20th generation after seeding in a 6-well plate (seeding density of 5 × 10^3^ cells/cm^2^). At intervals of 24 h, the cells were detached by trypsin-EDTA solution and counted with the automatic cell counter. Average cell number per well and standard deviation (SD) were considered to describe the population doubling level (PDL). At first, the population doubling (PD) was calculated according to the equation:

$$P.D.=\left(logN_{\text{t}}-logN_{0}/log2\right)$$

where N_t_ is the number of cells counted at the time point considered; and N_0_ is the number of cells seeded at *t* = 0 (Ferroni et al., 2019). Then, the PDL of every passage was determined by addition of the PD relative to that passage to the PD of previous passages (Marrelli et al., 2013).

Cell viability was also assessed at 8th, 14th, and 20th generations using the Apoptotic/Necrotic/Healthy Cells Detection Kit (Promokine, Cat No. PK-CA707-30018, Heidelberg, Germany) following the Manufacturer’s instructions. The detection of stained cells was performed with a DMR microscope (Leica, Wetzlar Germany).

To assess OA-IFP cells metabolic activity, the subcultures were treated with 3-(4,5-dimethylthiazol-2-yl)-2,5-dimethyltetrazolium bromide (MTT) (0.5 mg/mL) for 4 h, at 1, 2, 5, 7, and 14 days from seeding. Formazan precipitates were dissolved in 2-propanol acid (0.04 M HCl in 2-propanol) and optical density was measured at 570 nm, using a Microplate autoreader EL 13. Results were expressed as number of cells grown on seeded surface (Grandi et al., 2018). Determining cell number was allowed by the standard curve previously developed (Gerlier and Thomasset, 1986).

### Morphological Study

The OA-IFP stem cells and subcultures were observed by an optical microscope DM/IL (Leica, Wetzlar, Germany) equipped with a camera Nikon Digital Sight Ds-SMCc (Nikon Corporation, Tokyo, Japan). For ultrastructural studies, the cells were fixed with 2.5% glutaraldehyde (*Polysciences* Europe GmbH, Hirschberg an der Bergstrasse - Germany) in 0.1 M phosphate buffer and post-fixed with 1% osmium tetroxide (Agar Scientific Elektron Technology, United Kingdom) in 0.1 M phosphate buffer. After dehydration in a graded alcohol series, the samples were embedded in epoxy resin (Epoxy Embedding Medium Kit, Sigma-Aldrich, Switzerland) and processed as previously described (Stocco et al., 2018). Both 1% Toluidine blue staining and analysis by a Hitachi H-300 Transmission Electron Microscope (Hitachi, Krefeld, Germany) occurred.

### Stemness and Metabolic Gene Profile

Genes related to stemness (*TERT, REX1, SOX2*, *STAT3*, *NOTCH1*, *c-Myc*, *OCT-4*, *KLF4*, and *NANOG*) and metabolic activity (*CD38* and *CALR*) of OA-IFP stem cells were characterized by RT-qPCR. Cells from 8th passage at a sub-confluent state (i.e., 70–80%) were used to extract RNA with TRI Reagent^®^ solution (Zymo Research, United States). After RNA quantification using the NANODROP 2000 (Thermo Fisher Scientific, Waltham, MA, United States), gene expression study was carried out in a single-step reaction, using 12.5 ng of RNA, oligonucleotides (Thermo Fisher Scientific, Inc) listed in Table 2, qPCRBIO SyGreen 1-Step Go Lo-ROX (PCR Biosystems Ltd., London, United Kingdom) and Magnetic Induction Cycler (Bio Molecular Systems, Upper Coomera, QLD, Australia). To obtain statistical significance, three independent preparations were obtained to analyze target genes and *hypoxanthine phosphoribosyltransferase 1* (*HPRT1*) housekeeping marker listed in Table 2. For data analysis, the comparative Ct method (2^–Δ^ ^Ct^) was adopted. Human leukocytes isolated by a standard density gradient separation from healthy donors were used as control for the analysis of *CD38* and *CALR*.

**TABLE 2:** Oligonucleotides used for RT qPCR analysis.

	**Target gene**	**Sequence (5′–3′)**	**Reference sequence**	**Length (pb)**
*Hypoxanthine Phosphoribosyltransferase 1*	*HPRT1*	F: ATGGACAGGACTGAACGTCTTGCT	NM_000194.2	79 pb
		R: TTGAGCACACAGAGGGCTACAATG		
*CD38 molecule*	*CD38*	F: CTCAATGGATCCCGCAGTAAA	D_84276.1	145 pb
		R: CCTGGCATAAGTCTCTGGAATC		
*Calreticulin*	*CALR*	F: TCTCCCGATCCCAGTATCTATG	NM_004343.3	109 pb
		R: CATCGTTGGTGATGAGGAAGT		
*Telomerase reverse transcriptase*	*TERT*	F: ACATGGAGAACAAGCTGTTTGCGG	NM_198253.2	66 pb
		R: TGAGGTGAGGTGTCACCAACAAGA		
*Zinc finger protein 42*	*REX1*	F: TGGAGGAATTACCTGGCATTGACCT	NM_174900.3	105 pb
		R: AGCGATTGCGCTCAGACTGTCATA		
*SRY (Sex-determining Region Y)-Box 2*	*SOX2*	F: ACAACATGATGGAGACGGAGCTGA	NM_003106.3	191 pb
		R: TGGTAGTGCTGGGACATGTGAAGT		
*Signal transducer and activator of transcription 3*	*STAT3*	F: ATGGAAGAATCCAACAACGGCAGC	NM_213662.1	175 pb
		R: AGGTCAATCTTGAGGCCTTGGTGA		
*Notch receptor 1*	*NOTCH1*	F: TCAGGGTGTGCACTGTGAGATCAA	NM_017617.3	112 pb
		R: AGGTGCCGTTGTTAAAGCACTTGG		
*MYC proto-oncogene, bHLH transcription factor*	*c-Myc*	F: CTCCACACATCAGCACAACTA	D10493.1	79 pb
		R: TGTCCAACTTGACCCTCTTG		
*POU class 5 homeobox*	*OCT4*	F: TATGCAAAGCAGAAACCCTCGTGC	NM_002701.4	102 pb
		R: TTCGGGCACTGCAGGAACAAATTC		
*Kruppel-like factor4*	*KLF4*	F: TGAACTGACCAGGCACTACCGTAA	NM_004235.4	106 pb
		R: TCTTCATGTGTAAGGCGAGGTGGT		
*Nanog homeobox*	*NANOG*	F: CCCAAAGGCAAACAACCCACTTCT	NM_024865.2	106 pb
		R: AGCTGGGTGGAAGAGAACACAGTT		
*Collagen Type I Alpha 1 Chain*	*COL1A1*	F: CGATGGCTGCACGAGCTACAC	NM_000088.3	179 pb
		R: TGTCCTCATCCCTCTCATACA		
*Collagen Type II Alpha 1 Chain*	*COL2A1*	F: CGGGCAGAGGGCAATAGCAGGTT	NM_001844.4	127 pb
		R: CAATGATGGGGAGGCGTGAG		
*Collagen Type IX Alpha 3 Chain*	*COL9A3*	F: AATCAGGCTCTCGAAGCTCATAAAA	NM_001853.3	99 pb
		R: CCTGCCACACCCCCGCTCCTTCAT		
*Collagen Type X Alpha 1 Chain*	*COL10A1*	F: GAACTCCCAGCACGCAGAATCC	NM_000493.3	144 pb
		R: GTGTTGGGTAGTGGGCCTTTTATG		
*Secreted protein acidic and cysteine rich*	*SPARC*	F: TACATCGGGCCTTGCAAATA	J03040.1	121 pb
		R: CAGGTTGGGATGGAGGGAGTTTAC		
*Cortactin*	*CTTN*	F: GCAGAAGGATCGGATGGATAAG	BC008799.2	90 pb
		R: GCTTCGACAGGTACTGTCTTC		
*Glucose transporter 1*	*GLUT1*	F: GTGCTCCTGGTTCTGTTCTT	NM_006516.3	126 pb
		R: CTCGGGTGTCTTGTCACTTT		
*Glucose transporter 4*	*GLUT4*	F: GCCCTACGTCTTCCTTCTATTT	NM_001042.2	150 pb
		R: GGTTTCACCTCCTGCTCTAAA		

### Immunophenotyping

Sub-confluent (i.e., 70–80%) OA-IFP stem cells from 8th generation were analyzed by single staining with FACSCanto II Flow cytometer (BD Biosciences, San Diego, CA, United States) at 24 h after seeding, using anti-human antibodies (Table 3). FCM analysis were performed to evaluate the expression of markers related to mesenchymal stem cell immunophenotype, hematopoietic lineage commitment, enzyme/signaling molecules, cell–matrix adhesion and cytokine/growth factor receptors. For each marker, data from three experimental replicas were analyzed with FlowJo^TM^ software (BD) and then were reported as mean percentage of positive cells. Samples treated with only secondary antibodies or isotype control antibodies (Table 3) were considered as controls. No exclusion of dead cells from analysis was performed.

**TABLE 3:** Antibodies used for flow cytometry analysis.

	**Ref. number**	**Manufacturing company**
**Primary antibodies**		
PE anti-human CD34 (BI-3C5)	sc-19621	Santa Cruz Biotechnology, Inc
Mouse anti-human CD39	sc-65262	Santa Cruz Biotechnology, Inc
PE anti-human CD44 (H-CAM F-4)	sc-9960	Santa Cruz Biotechnology, Inc
PE anti-human CD45 (2D1)	sc-1187	Santa Cruz Biotechnology, Inc
PE anti-human CD73	344004	BioLegend, Inc
FITC anti-human CD90 (aTHy-1A1)	sc-53456	Santa Cruz Biotechnology, Inc
PE anti-human CD117 (c-Kit Ab81)	sc-13508	Santa Cruz Biotechnology, Inc
PE anti-human CD105 (Endoglin P3D1)	sc-18838	Santa Cruz Biotechnology, Inc
PE mouse anti-human HLA-DR (L243)	sc-18875	Santa Cruz Biotechnology, Inc
A350 anti-human IL1R1	bs-2594R	Bioss
Rabbit anti-human IL6R	AB-83485	Immunological Sciences
APC anti-human FGFR2	FAB 684A	R&D Systems, Inc
PE anti-human PDGFRβ	558417	BD Biosciences
FITC anti-human VEGFR2/KDR	FAB 357F	R&D Systems, Inc
PE anti-human CD95 (Fas)	555674	BD Biosciences
APC anti-human CD178 (FasL)	564262	BD Biosciences
**Secondary antibodies**		
AF488 goat anti-mouse	A32723	Invitrogen
AF488 goat anti-rabbit	A32731	Invitrogen
**Isotype controls**		
PE isotype control	sc-2855	Santa Cruz Biotechnology, Inc
PE isotype control	400114	BioLegend, Inc
PE isotype control	554680	BD Biosciences
FITC isotype control	sc-2855	Santa Cruz Biotechnology, Inc
FITC isotype control	IC002F	R&D Systems, Inc
APC isotype control	IC002A	R&D Systems, Inc
APC isotype control	555751	BD Biosciences
A350 isotype control	bs-0295P	Bioss

### CD45 and HLA-DR Double Staining

To confirm FCM data, a CD45 and HLA-DR double staining was performed on OA-IFPs tissue sections from samples used for stem cells isolation.

Osteoarthritic infrapatellar fat pad specimens were fixed in 10% formalin in PBS, paraffin-embedded and cut into 4 μm-thick sections. After deparaffinization, the sections were treated with EDTA (pH = 8; for Mouse Anti-Human-CD45) and citrate buffer (pH = 6; for Mouse Anti-Human HLA-DR) in microwave oven heating. After washing in PBS, the specimens were incubated with Dual Endogenous Enzyme block (ready to use – Dako, Milan, Italy) to assure endogenous peroxidase and alkaline phosphatase inhibition, rinsed in PBS, blocked in 0.2% BSA (Bovine Serum Albumin) and incubated with Mouse Anti-Human CD45 1:100 (Biocare CM016) overnight at 4°C. After washing, the samples were incubated with the secondary antibody (HRP-conjugated Goat Anti-Mouse – Jackson ImmunoResearch, Cambridgeshire, United Kingdom) and the reaction was detected with diaminobenzidine, Dako (brown stain). Thus, the sections were rinsed in PBS and incubated with Mouse Anti-Human HLA-DR 1:100 (Clone TAL.1B5 – Dako) overnight at 4°C. The slides were subsequently rinsed in PBS, covered with Mouse AP Polymer and HLA-DR was visualized with GBI-Permanent Red solution (Red stain). The samples were counterstained with Harris modified hematoxylin (Sigma, Milan, Italy) before mounting.

### *In vitro* Differentiative Response to Microenvironmental Stimuli

#### Native Plasticity

IFP specimens from OA patients are demonstrated to contain high level of fibrosis (Favero et al., 2017) and to be characterized by cells endowed with chondrogenic and osteogenic potential (Hindle et al., 2017). Thus, a gene expression study by qPCR was carried out using subcultures under resting conditions and at a sub-confluence state, in order to define the expression of extracellular-matrix related proteins of cartilage/bone tissue (*COL1A1, COL2A1, COL9A3, COL10A1, SPARC)*, pro-fibrogenic (*CTTN*) markers and glucose transporters (*GLUT1, GLUT4*) as above reported, using oligo primers listed in Table 2.

#### *In vitro* Adipogenic Differentiation

Osteoarthritic infrapatellar fat pad stem cells ability to be used for creating an *in vitro* model of adipose tissue, resembling the *in vivo* environment, was verified. Thus, OA-IFP stem cells were seeded in a 24 well plate (5 × 10^3^ cells/cm^2^) and cultured in proliferative medium for 3 days. To mimic the IFP environment, sub-confluent cells where then exposed to adipogenic soluble stimuli: insulin (10 μg/mL), dexamethasone (1 μM); indomethacin (60 μM), 3-isobutyl-1-methyxantine (0.5 mM) (all from Sigma-Aldrich) in basal medium (DMEM HG, 10% FBS, 1% P/S); cells under resting conditions were used as controls. At days 7 and 14, the cells were fixed with a 10% formalin solution (Sigma-Aldrich) and stained with Oil Red O (5 mg/ml in isopropanol) according to standard protocol. After mounting with glycerol, all samples were analyzed by a Leica DMR microscope endowed with a Nikon Digital Sight Ds-SMCc camera (Nikon Corporation).

#### Adaptive Response to Vascular Related Signal and to Mechanical Force

In order to evaluate the response of OA-IFP stem cells to pathological environment, an *in vitro* system was set up. In details, endothelial medium [EBM^TM^-2 medium (Lonza Group AG, Switzerland), 5% FBS, 1% P/S] enriched of angiogenic factors [vascular endothelial growth factor (VEGF)], human basic fibroblast growth factor (hFGF-b), epidermal growth factor (EGF), insulin-like growth factor-1 (IGF-1), heparin, ascorbic acid was used in combination with stiff (glass coverslip) or soft (BD Matrigel diluted 1:10 in EBM^TM^-2 medium) substrates. The soluble factors were used to simulate the vascular-related signals, while soft or stiff substrates were employed to reproduce mechanical loading forces. It is expected that cells may respond differently according to the specific stimuli they exposed to. OA-IFP stem cells seeded on plastic surface of 24 well plate (5 × 10^3^ cells/cm^2^) and cultured in proliferation medium represented the control-group.

At 3 and 7 days, the cultures were observed by a Leica DMR microscope endowed with a Nikon Digital Sight Ds-SMCc camera (Nikon Corporation) and pictures at 10x were processed through ImageJ software as described by Guidolin et al., 2004 to detect the branches (i.e., number and length) and the branching points.

### Statistical Analysis

Statistical analysis was performed with GraphPad Prism 6.0 (GraphPad Software, Sand Diego, CA, United States). Data referring to age and body mass index of the patients were expressed as median and interquartile range. All experimental data were expressed as average of three different experiments (technical replicates) ±Standard Deviation (SD) referring to nine patients. Statistical significance was determined by unpaired *t*-test and results were considered significant when *P* < 0.05.

## Results

### Cell Isolation and Morphology in Culture

**FIGURE 1:**
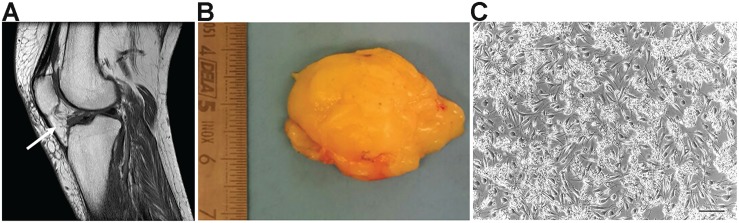
Osteoarthritic infrapatellar fat pad (OA-IFP) and derived cells. **(A)** Magnetic resonance imaging of the knee in sagittal section, showing the infrapatellar fat pad (IFP – white arrow) before total knee arthroplasty(TKA). **(B)** Gross appearance of the IFP after surgical excision and before tissue enzymatic digestion for cells isolation. **(C)** Representative optical microscope image of the IFP stem cells at P0 in culture. P, passage. Scale bar, 100 μm.

IFP tissue samples were obtained from both male (*n* = 3) and female (*n* = 6) patients undergoing TKA (Figures 1A,B). The samples showed the presence of a highly vascularized synovial membrane, which was removed before tissue processing in order to obtain only IFP-native stem cells. Tissues had a fibrous consistency; in particular, a certain resistance during fragmentation was encountered in the external layer. On the contrary, the inner layer was less fibrotic.

The whole isolation process was completed in 14 h, considering the over-night incubation period in the presence of the digesting enzyme collagenase B. At the end of the isolation protocol, the cells were resuspended in the proliferation medium and seeded at high density. It was not possible to accurately determine the mean total number of cells extracted from each sample due to the presence of extracellular matrix (ECM) fragments in suspension hindering the total cell count. However, high cell density as well as the maintenance of matrix residues were necessary to optimize cell adhesion and survival in monolayer culture (Figure 1C); tissue debris were then easily removed by subsequent changes of medium and passages in culture.

### Long-Term Proliferation Potential and Metabolic Activity of OA-IFP Cells

**FIGURE 2:**
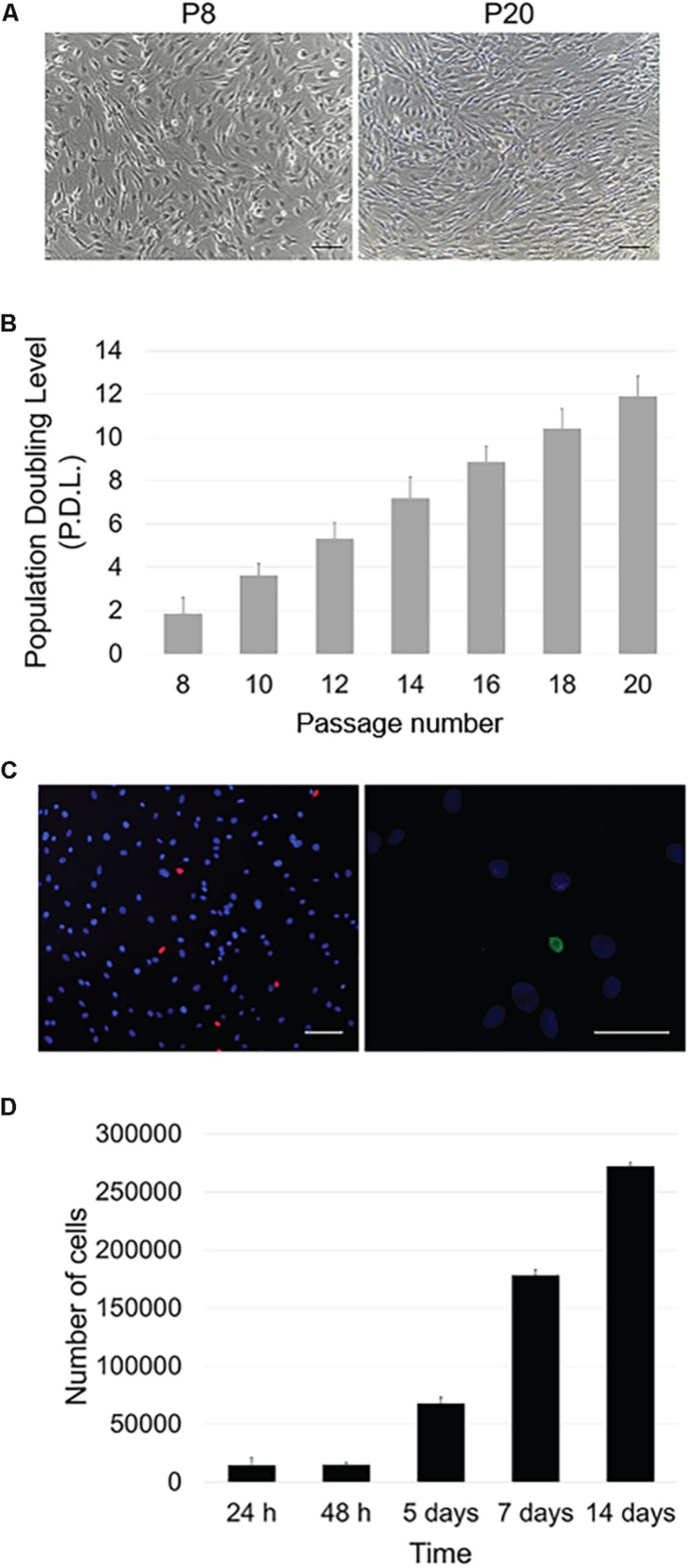
Proliferative potential and metabolic activity. **(A)** Optical microscope images of OA-IFP cells in proliferative medium at P8 and P20. P: passage. Scale bars, 100 μm. **(B)** Population Doubling Level (PDL) calculated throughout 12 generations; cells performed 11.9 ± 8.3 population doublings. **(C)** Vitality/necrosis/apoptosis test after treatment with fluorescent dyes. Blue dye stained viable cells while red elements corresponded to necrotic cells (scale bar, 100 μm); apoptotic cells were colored in green (scale bar, 50 μm). The images are representative of cells at 8th, 14th, and 20th generations. **(D)** Metabolic activity (MTT assay) of OA-IFP cells from 24 h to 14 days in culture. For the panels **(B,D)**, results are the average of 3 technical replicates each referring to 9 patients.

In culture, OA-IFP stem cells showed a typical spindle-like morphology which was maintained along passages in culture (Figure 2A). The proliferation rate of OA-IFP cells was evaluated in 12 consecutive generations to assess their ability to sustain long-term culture without reaching senescence. As reported by Figure 2B, from passage 8th to 20th the stem cells had performed 11.9 ± 8.3 population doublings. Vitality was also confirmed by specific staining using fluorescent dyes (Figure 2C).

MTT assay was used to assess OA-IFP stem cells metabolic activity also demonstrating a progressive cell growth at different endpoints (Figure 2D). From 24 to 48 h after seeding, the cell number varied moderately due to an initial slow expansion. Since day 5th, the metabolic activity and the cell number increased and the same trend was observed at day 7.

At 14 days from seeding, the contact inhibition between cells at confluence influenced the metabolic activity resulting in a slower cell growth.

### Morphology and Metabolic Stress-Related Markers

**FIGURE 3:**
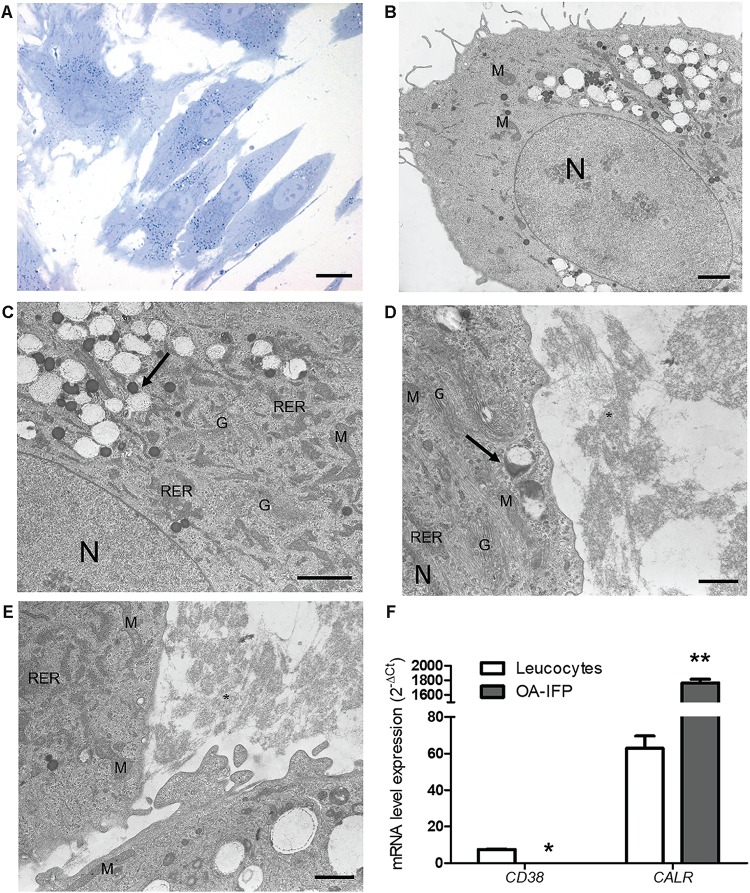
Ultrastructural characterization of OA-IFP stem cells. Toluidine Blue staining images **(A)** and TEM micrographs **(B–E)** of OA-IFP cells at P8 in culture. Black arrows in **(C)** and **(D)** show electron dense lysosomes and empty vesicles. Black asterisk in **(E)** shows secretion material resembling collagen fibrils outside the cytoplasm. M, mitochondria; N, nucleus; G, golgi apparatus; RER, rough endoplasmic reticulum. Scale bars, **(A)** 20 μm; **(B)** and **(C)** 2 μm; **(D)** and **(E)** 1 μm. **(F)** mRNA expression levels of CD38 and CALR in OA-IFP stem cells compared to human leukocytes (^∗^*P* < 0.5; ^∗∗^*P* < 0.01). For panel **(F)**, results are the average of 3 technical replicates referring to 9 patients.

Toluidine blue staining displayed cells with a regular appearance and deeply blue-stained vesicles distributed in perinuclear areas (Figure 3A). At TEM analysis, ultrastructural cell constituents like mitochondria, rough endoplasmic reticulum, Golgi apparatus, secretion vesicles were clearly identified in the cytoplasmic area surrounding cell nucleus which appeared voluminous and bordered by a thin and regular membrane (Figures 3B,C). Lysosomes were also recognized within the cytoplasm appearing as rounded formations of electron-dense material corresponding to those deeply stained with toluidine-blue in Figure 3A. Interestingly, TEM analysis also allowed to discriminate apparently empty round vesicles surrounding the dense ones. Such non-electron dense elements showed the presence of a thin honeycomb-like structure; in some contact areas, a partial fusion between the external membranes of the two vesicles types occurred. Filamentous and disorganized cell secretion products were localized in proximity to cell membrane (Figures 3D,E).

From the metabolic point of view, the analysis by qPCR on OA-IFP stem cells *versus* leukocytes, showed null-expression of mRNA for *CD38* and significantly (*P* < 0.01) higher levels of *calreticulin* gene (Figure 3F).

### Multipotency of OA-IFP Stem Cells

**FIGURE 4:**
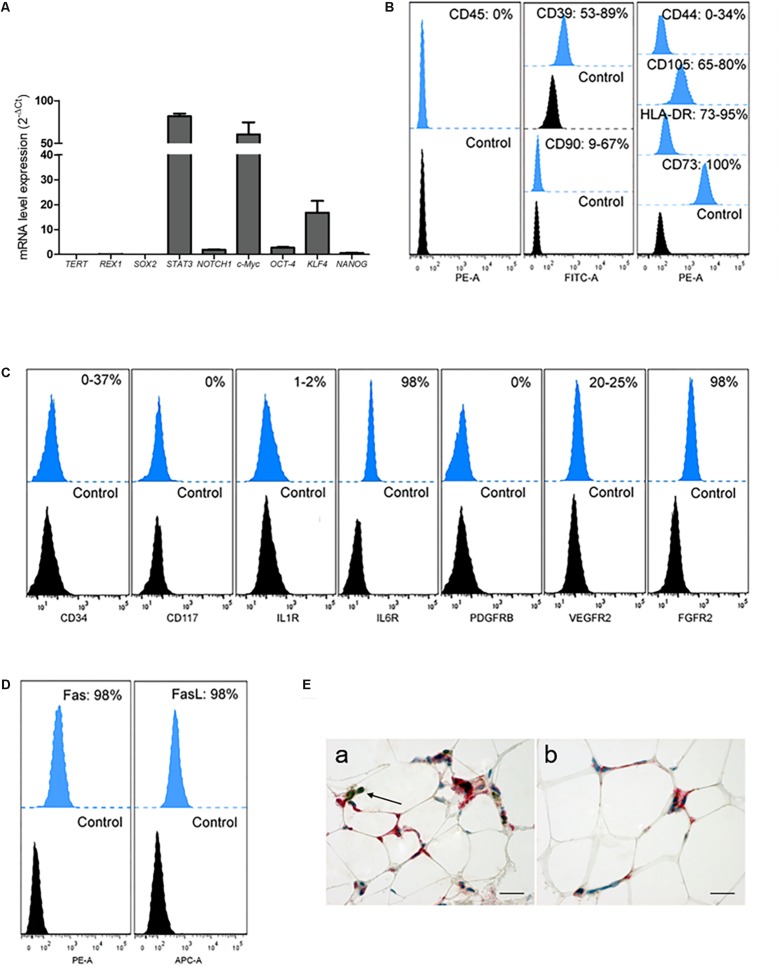
Analysis of OA-IFP by qPCR, FCM and immunohistochemistry. OA-IFP subcultures from 8th generation and under proliferative conditions were analyzed at a sub-confluence state for stemness **(A,B)** or cellular responsivity markers **(C,D)** by qPCR **(A)** and FCM [FacsDiva and FlowJo software **(B,D)**] **(B–D)** where the histograms show the mean percentage of positive cells; samples treated with only secondary antibodies or isotype control antibodies were used as references. Three technical replicates for each sample were analyzed. **(E)** Double immunohistochemical analysis of OA-IFP tissue samples showing cellular elements positive to HLA-DR (red) and negative to CD45 (brown). Lymphocytic elements, positive to only CD45 (brown), were considered as an internal control of the method (black arrow, **E,a**) (Scale bar: 25 μm). FCM, flow cytometry analysis.

During *in vitro* short and prolonged expansion, the active mRNA expression of *NANOG, c-Myc, NOTCH1, KLF4* and *STAT3* (Figure 4A) proved a multipotential grade of OA-IFP stem cells. By FACS analysis (Figures 4B,C) OA-IFP populations evidenced an almost homogenous immunophenotypic profile. In particular, the samples showed to be over 90% positive for the mesenchymal stem cell marker CD73, FGFR2, and IL6R suggesting both immunomodulatory activity and cellular responsivity to IFP- and OA-microenvironmental secreted factors. The cellular multipotency was confirmed by the detection of CD105, CD90, and CD44, whose variable expression among donors was hypothesized to be related to the age or inflammation state of patients. Due to their significant expression of HLA-DR (≥90%), Fas/FasL (≥98%), CD39, VEGFR2, and, only in some cases, of CD34, OA-IFP stem cells could represent a cellular compartment primed by chronic inflammatory conditions (Figures 4C,D). The null expression of CD45, CD117, and PDGFRβ excluded their possible derivation from hematopoietic/endothelial progenitors or perivascular stem cell niche (Figures 4B,C). Double immunohistochemistry on OA-IFP tissue samples confirmed the presence of cellular elements (Figure 4E) simultaneously positive to HLA-DR and negative to CD45 (Figure 4C). Taken together, our collected data evidenced distinctive immunophenotypic properties of OA-IFP compared to other multipotent populations isolated from IFP.

### Differentiation Potential and Response to Environmental Stimuli of OA-IFP Stem Cells

**FIGURE 5:**
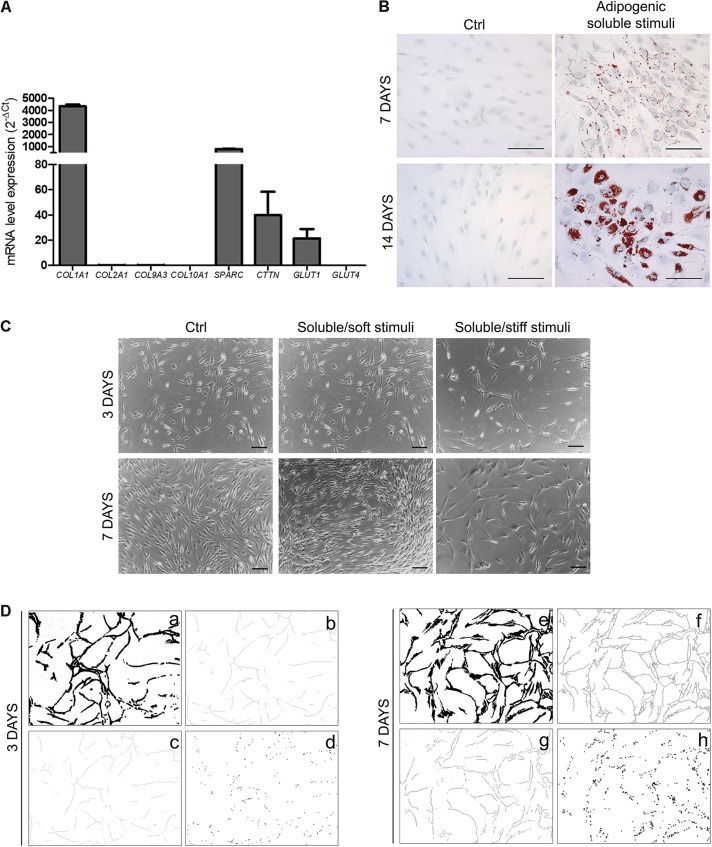
OA-IFP stem cells responsiveness to environmental stimuli. **(A)** qPCR analysis of mRNA expression levels of *COL1A1*, *SPARC*, *GLUT1*, and *CTTN* gene on OA-IFP stem cells at a sub-confluence state; null-expression of *GLUT4*, *CTTN*, *COL2A1*, *COL9A3*, *COL10A1* mRNAs was detected. **(B)** Response of OA-IFP stem cells to adipogenic stimuli. After 7 and 14 days from stimulation, the cells showed cytoplasmic red-stained lipid droplets; scale bars, 100 μm. **(C)** Response of OA-IFP stem cells to soluble (endothelial medium enriched of angiogenic factors) and mechanical stimuli (stiff and soft support) at 3 and 7 days from stimulation. OA-IFP stem cells seeded on plastic surface and cultured in proliferation medium represented the control-group; scale bars, 100 μm. **(D)** Image processing steps of images referring to OA-IFP stem cells cultured on stiff support, at 3 and 7 days from stimulation. After edge detection and threshold binary tools **(a,e)** the skeletons were calculated **(b,f)**, before the analysis of branches (i.e., number and length) **(c,g)** and branching points **(d,h)**. The processed images highlight the formation of the cord-like structures. For panel **(A)**, results are the average of 3 technical replicates for each sample referring to 9 patients.

Under resting conditions, OA-IFP stem cells also demonstrated to have a differentiation potential into chondrogenic, and osteogenic lineages producing mRNAs of *COL1A1, SPARC*, and *GLUT1*; in addition, the significant expression of *cortactin*/*CTTN* gene suggested that the differentiation of OA-IFP stem cells might be regulated by mechano-transduction (Figure 5A). When treated with adipogenic stimuli, the cells acquired distinct functionality as demonstrated by the cytoplasmic accumulation of lipid droplets (Figure 5B). Moreover, combining specific soluble (endothelial medium enriched with angiogenic factors) and mechanical stimuli (stiff or soft), OA-IFP cells showed different behaviors (Figure 5C). On glass (i.e., stiff support) cells acquired an elongated morphology, describing cord-like structures; cell density remained comparable at both end-points (3 and 7 days). On Matrigel (i.e., soft support), an initial response similar to that previously described occurred at day 3. Thereafter cell density increased and the network was no more identifiable. In contrast, the control group did not show any specific cell orientation nor reduction in proliferation.

After processing the images referring to OA-IFP stem cells cultured on stiff support (Figure 5D), it was observed a progressive increase in the number and length of the branches (+59.25 and +76.49%, respectively) and in the number of the branching points (+55.28%). The number of the branches increased from 132.43 ± 11.89 (day 3) to 223.50 ± 13.05 (day 7) per square unit (μm^2^); the length of the branches (expressed as linear value - μm) increased from 8830.86 ± 639.29 (day 3) to 11543.92 ± 1156.96 (day 7). A variation in the number of the branching points was also observed, from 87.70 ± 11.19 to 158.63 ± 13.44 per square unit (μm^2^) at day 3 and 7, respectively.

## Discussion

To date, IFP stem cells have been proposed as an alternative to subcutaneous adipose-derived stem cells and an attractive cell source for cartilage regeneration due to their ability for differentiation into chondrocytes, minor immunological rejection and constitutive immunomodulatory properties (Felimban et al., 2014; Ye et al., 2014, 2016; Tangchitphisut et al., 2016; Arora et al., 2017; Sun et al., 2018; Radhakrishnan et al., 2019). Inflammatory processes, microenvironmental changes, chemical factors and physical cues evoke a tissue adaptive and protective response acting in concert on immune cells and/or endogenous stem cells. In contrast to López-Ruiz et al. (2013), in this study we have demonstrated that in OA, IFP-derived stem cells might be reprogramed by the inflammatory environment preserving multi-differentiative plasticity. Thus, they might exert a protective role in counteracting mechanical overloading through deposition of collagenous matrix.

In accordance with the peculiar clinical OA framework, the patients enrolled in the study suffered from obesity, hypertension, dyslipidemia and type 2 diabetes, that are all conditions of chronic stress (Courties et al., 2017) affecting joint structures, including IFP and its cellular compartments.

Showing adherent and spindle-like morphology, OA-IFP stem cells were proved to have a high metabolic activity and a population doubling time of 42.7 ± 3.8 h likely adipose stem cells (Peng et al., 2008).

As previously described by López-Ruiz et al. (2013), TEM analysis on OA-IFP stem cells evidenced a cytoplasm rich in transport/storage vesicles and deeply stained/electron dense structures, rough endoplasmic reticulum and mitochondria. Higher magnifications also suggested the presence of autolysosomes, a product of fusion of lysosomes (dark, electron dense vesicles) and autophagosomes (large, white vacuoles) that are deputed to autophagy (Revuelta and Matheu, 2017; Boya et al., 2018), a process that have been suggested to have a role in OA development (Deng et al., 2019). In fact, there is consensus in claiming that autophagy can be triggered by a variety of internal or external stimuli as well as hypoxia, Reactive Oxygen Species (ROS) and accumulation of unfolded proteins, which are all conditions typically encountered in OA (Hughes et al., 2017).

Acting like scavengers, autolysosomes catabolize undesirable components to provide energy and essential building materials to maintain cellular homeostasis. Hence, according to these assumptions, OA-IFP stem cells likely switch-on this mechanism to counteract the disease-related stress condition.

Stem cells take advantage from glycolysis to gain energy and regulate the undifferentiated status (Zhang et al., 2016), while mitochondrial function is linked to stem cells activation. The significant amount of mitochondria (Bravo et al., 2011) (as shown also for OA-IFP stem cells by TEM), correlated with the high expression levels of calreticulin gene (Ní Fhlathartaigh et al., 2013). Calreticulin was assumed as an indicator of cell stress condition, activated status and a switched nicotinamide adenine dinucleotide (NAD^+^)-dependent energy metabolism. In OA, it has been reported that the unbalanced biomechanics cause a self-perpetuating cycle of low-grade damage (Scanzello et al., 2008) that, in turn, leads to the production of damage-associated molecular patterns. Cartilage ECM breakdown products, fibronectin and hyaluronic acid (Okamura et al., 2001; Termeer et al., 2002; van Lent et al., 2012) and intracellular alarmins [HMGB1 (Li et al., 2011), S100 proteins (van Lent et al., 2012)] signal on synovial macrophages, synoviocytes, or chondrocytes promoting the local production of numerous inflammatory mediators [IL-1β (He et al., 2002), VEGF, TNFα, IL-6 (Ushiyama et al., 2003), PDGFs (Pohlers et al., 2006)]. Moreover, regulatory mechanisms involved in controlling NAD^+^ levels have been demonstrated to be affected in OA because of an increased expression of IL-β and a compromised activity of NADases (Kobayashi et al., 2017). Thus, endoplasmic reticulum stress and highly expressed vacuoles in concert with the expression of prognostic OA marker IL-1βR1 highlight the occurrence of a strict correlation between OA-IFP biology and the inflammatory microenvironment (Daheshia and Yao, 2008). In addition, CD38/NADase, that is commonly up-regulated to reduce glycolytic and mitochondrial metabolism in OA (Chang et al., 2014), is not expressed in OA-IFP stem cells suggesting an impairment of the regulative mechanisms responsible of metabolic activity. Compared to other IFP-derived multipotent cells (Wickham et al., 2003; López-Ruiz et al., 2013; Garcia et al., 2016; Courties et al., 2017; do Amaral et al., 2017; Hindle et al., 2017), the OA-IFP stem cells investigated in this study have demonstrated not only distinct functional abilities but also characteristic immunophenotypic properties. Differences may probably be attributable to peculiarities in the patients cohort (i.e., age and disease severity) and/or in the isolation protocol (i.e., digestion times and processing phases of the digested tissue). In particular, the population showed the expression of *STAT3*, *NOTCH1, c-Myc, OCT-4, KLF4*, and *NANOG*, suggesting to have high self-renewal potential (Graf and Stadtfeld, 2008) while confirming their inflammation-activated state through the expression of HLA-DR, Fas and FasL (Hashimoto et al., 1998; Kim et al., 2000; Wickham et al., 2003), as already observed in patients affected by OA. These data are in line with the hypothesis that the inflammatory nature of the fat pad could condition endogenous stem cell niche upregulating HLA-DR (Wickham et al., 2003), but not promoting the expression of costimulatory molecules (Rübenhagen et al., 2012; Greene and Loeser, 2015). Due to the expression of CD34, a derivation of OA-IFP stem cells from adipose tissue (Bourin et al., 2013) or vascular endothelial compartment (Traktuev et al., 2008) can be hypothesized. While a derivation from perivascular niche is unlikely, as the pericyte marker PDGFRβ expression was lacking. Moreover, the expression of adhesion molecules (CD44, CD105), receptors for growth factors (VEGFR2, FGFR2) and cytokines (IL1R, IL6R) together with mRNA transcript of *cortactin* (*CTTN*) suggested a high level of microenvironmental responsiveness, further supporting the hypothesis that they are an endogenous subpopulation primed by the inflammatory milieu and able to switch to adaptive fibrogenic reprograming under unbalanced mechanical stimuli or dysregulated mechano-transduction. It is known that extracellular ATP levels increase in response to tissue-disturbing events, including inflammation, hypoxia or ischemia (Di Virgilio and Adinolfi, 2017). Based on our data, despite expressing a typical anti-inflammatory signature mediated by CD39 and CD73 (Eltzschig, 2013), OA-IFP stem cells could be limited *in vivo* to control the complex immune response related to OA. OA-IFP tissue is characterized by highly vascularized adipose lobuli separated by thickened septa (Favero et al., 2017; Fontanella et al., 2017) that, in turn, could prime endogenous stem cell populations. In our study, OA-IFP stem cells responded to adipogenic stimuli by accumulating lipid cytoplasmic droplets; in parallel, when stimulated with combined endothelial and mechano-transduction factors, net-like structures were observed on stiff-surface while a disorganized cell distribution was evidenced on soft matrix. Thus, the formation of net-like structures correlated with the high expression level of *COL1A1* gene may suggest the potential involvement of OA-IFP stem cells to exert *in vivo* a protective response against mechanical overloading by thickening the fibrous septa. This adaptive-behavior is supported by significant body of evidence in the literature; the cells, including mesenchymal stem cells, can sense mechanical stress and soluble stimuli and translate such information in adaptive responses including differentiation, changes in morphology and cytoskeletal dynamics, also varying gene expression (McBeath et al., 2004; Chiquet et al., 2007, 2009). In OA, it is conceivable that stem cells expressing ectonucleotides and adenosine deaminase like OA-IFP cells could contribute *in vivo* to the generation of extracellular adenosine, that is a recognized checkpoint mediator for immune suppression (English and Mahon, 2011). Based on the evidence that OA-IFP stem cells have a low expression of *CD38/NADase*, we speculated that they could not be effective to counteract NAD^+^-mediated inflammation which is an important signature of OA.

Future pre-clinical studies based on OA-IFP stem cells will be useful to highlight their active or passive role in chronic OA-inflammation. In particular, functional studies developed in animal models of OA with adequate control-population will allow for a more specific understanding of OA-IFP stem cells behavior and role.

## Data Availability Statement

The datasets generated for this study are available on request to the corresponding author.

## Ethics Statement

This study was performed after receiving Institutional Review Board approval (CESC Code: 4510/AO/18). The patients/participants provided their written informed consent to participate in this study.

## Author Contributions

ES, AnP, RDL, RDC, and VM: conception and design of the study. ES, SB, MP, EB, LP, and AsP: data acquisition. ES, RR, MR, MF, PR, RDL, and VM: data analysis and interpretation. ES and MP: drafting the article. All authors aided in revising this manuscript for intellectual content and approved the final version to be published.

## Conflict of Interest

The authors declare that the research was conducted in the absence of any commercial or financial relationships that could be construed as a potential conflict of interest.
